# Basal Dendritic Morphology of Cortical Pyramidal Neurons in Williams Syndrome: Prefrontal Cortex and Beyond

**DOI:** 10.3389/fnins.2017.00419

**Published:** 2017-08-11

**Authors:** Branka Hrvoj-Mihic, Kari L. Hanson, Caroline H. Lew, Lisa Stefanacci, Bob Jacobs, Ursula Bellugi, Katerina Semendeferi

**Affiliations:** ^1^Department of Anthropology, University of California, San Diego San Diego, La Jolla, CA, United States; ^2^Neuroscience Program, Colorado College Colorado Springs, CO, United States; ^3^Laboratory for Cognitive Neuroscience, The Salk Institute for Biological Studies La Jolla, CA, United States; ^4^Kavli Institute for Brain and Mind, University of California, San Diego San Diego, La Jolla, CA, United States

**Keywords:** dendritic morphology, Williams syndrome, neurodevelopmental disorders, Golgi, dendrite

## Abstract

Williams syndrome (WS) is a unique neurodevelopmental disorder with a specific behavioral and cognitive profile, which includes hyperaffiliative behavior, poor social judgment, and lack of social inhibition. Here we examined the morphology of basal dendrites on pyramidal neurons in the cortex of two rare adult subjects with WS. Specifically, we examined two areas in the prefrontal cortex (PFC)—the frontal pole (Brodmann area 10) and the orbitofrontal cortex (Brodmann area 11)—and three areas in the motor, sensory, and visual cortex (BA 4, BA 3-1-2, BA 18). The findings suggest that the morphology of basal dendrites on the pyramidal neurons is altered in the cortex of WS, with differences that were layer-specific, more prominent in PFC areas, and displayed an overall pattern of dendritic organization that differentiates WS from other disorders. In particular, and unlike what was expected based on typically developing brains, basal dendrites in the two PFC areas did not display longer and more branched dendrites compared to motor, sensory and visual areas. Moreover, dendritic branching, dendritic length, and the number of dendritic spines differed little within PFC and between the central executive region (BA 10) and BA 11 that is part of the orbitofrontal region involved into emotional processing. In contrast, the relationship between the degree of neuronal branching in supra- versus infra-granular layers was spared in WS. Although this study utilized tissue held in formalin for a prolonged period of time and the number of neurons available for analysis was limited, our findings indicate that WS cortex, similar to that in other neurodevelopmental disorders such as Down syndrome, Rett syndrome, Fragile X, and idiopathic autism, has altered morphology of basal dendrites on pyramidal neurons, which appears more prominent in selected areas of the PFC. Results were examined from developmental perspectives and discussed in the context of other neurodevelopmental disorders. We have proposed hypotheses for further investigations of morphological changes on basal dendrites in WS, a syndrome of particular interest given its unique social and cognitive phenotype.

## Introduction

The prefrontal cortex (PFC) consists of a number of distinct cytoarchitectonic areas that are part of neural systems subserving higher order cognitive and emotional functions. One aspect of microstructural neuroanatomy that has received attention in PFC and in other cortical areas is the dendritic morphology of pyramidal neurons –specifically, the relationship with other areas outside PFC, variation between distinct PFC areas underlying different aspects of information processing, and variation across cortical layers within the same cytoarchitectonically defined PFC area. Investigations of pyramidal neurons, as the most common neuronal morphotype in the cortex (DeFelipe and Fariñas, 1992; DeFelipe et al., 2002), provide insights into the units forming the basis of cortical microcircuitry within a functional area, their response to various environmental inputs (i.e., plasticity; reviewed by Hanson et al., 2014), and illuminate the neuroanatomical substrates underlying various disorders in an evolutionary and developmental perspective.

Studies of pyramidal neurons in typically developed controls (TD) suggest that the length, number of branches, and number of dendritic spines on basal dendrites of cortical pyramidal neurons differ across functionally distinct cortical areas in adults (Jacobs et al., 2001). Dendrites on pyramidal neurons in high-integration areas, such as BA 10, are typically longer, more branched, and have more dendritic spines than neurons in cortical areas devoted to a specific modality, like areas in the motor, sensory or visual cortex. Basal dendrites in BA 11 neurons are shorter, less branched and less spinous than in BA 10, differing only slightly from the primary somatosensory cortex (BA 3-1-2; Jacobs et al., 2001), the area with the least complex basal dendritic morphology. Primary motor cortex (BA 4) and secondary visual area (BA 18) are intermediate between BA 3-1-2 and BA 10, aligning more closely with the primary processing areas than with the high-integration ones (Jacobs et al., 1997, 2001). Basal dendritic morphology has also been shown to vary across layers within the same area—in PFC (BA 9), studies comparing layers II/III to V/VI in TD identify the basal dendrites of neurons in layer III as longer than those observed in infragranular layers (Petanjek et al., 2008), although some variation is present.

Developmentally, PFC is distinct from the rest of the cortex, with some aspects of its anatomy (e.g., dendritic spines) not reaching maturity until well into the third decade of life (Petanjek et al., 2011). Patterns similar to those described in neuroanatomical studies have been supported by gene expression analyses of the developing brain, suggesting prolonged activity of genes involved into maturation of synapses in PFC (Somel et al., 2009; Liu et al., 2012). This prolonged period of plasticity could potentially make PFC more prone to modifications in neurological disorders (Harris et al., 2009; Penzes et al., 2011). In various neurological disorders—ranging from schizophrenia, autism spectrum disorder (ASD), to chromosomal aberrations (Armstrong et al., 1998; Garey et al., 1998; Glantz and Lewis, 2000; Hutsler and Zhang, 2010)—PFC areas tend to display differences between the affected individuals and TDs. In neurological disorders, the organization of basal dendrites on pyramidal neurons—dendritic length, branching, number and organization of dendritic spines—is often compromised compared to TD subjects. Of particular interest is dendritic morphology in neurodevelopmental disorders characterized by an early onset of symptoms, specific cognitive impairments, and associated with specific genetic etiology, because they allow for the examination of the interplay between development, neuroanatomy, and genetics. Examples include trisomies (Down syndrome, Patau syndrome, Edwards syndrome), Rett syndrome (RTT), and ASD. In each of the above disorders, differences with TD subjects appear postnatally (Marin-Padilla, 1972, 1976; Jay et al., 1990) and dendritic morphology varies according to cortical area and cortical layers, suggesting that distinct aspects of dendritic morphology may be compromised in each disorder (but see also Kaufmann and Moser, 2000 for consistent findings of dendritic spine anomalies across disorders). In each of the disorders where PFC is included in the analysis, PFC areas typically exhibit compromised dendritic morphology, with differences appearing to be area- and layer-specific.

Williams syndrome (WS) is a rare disorder caused by the hemideletion of ~25 genes on chromosome 7 and characterized by an unusual sociability and preservation of certain linguistic aspects, coupled with a compromised spatial and general cognition (Bellugi et al., 1999, 2000). WS phenotype can thus be contrasted with ASD, especially since duplication on the WS deleted region on chromosome 7 has been implicated in some cases of ASD (Berg et al., 2007; Sanders et al., 2011). Unlike ASD, WS has a very specific set of affected genes and related phenotypic expression, which makes this disorder most suitable for investigations of the relationship among genes, brain, and behavior. Given the importance of PFC in social behavior, studying WS neuroanatomy allows to examine the possibility that changes in human social behavior can be traced to compromised PFC cortical microcircuitry. Here, we examined pyramidal neuron morphology in rare postmortem tissue from two WS individuals. We focused on the morphology of basal dendrites in supra- and infragranular layers in areas BA 10 and BA 11 in PFC and selected motor, somatosensory, and visual (MSV) areas—the primary motor (BA 4), primary somatosensory (BA 3-1-2), and secondary visual cortex (BA 18)—to examine whether PFC is differentially affected in comparison to other cortical regions.

More specifically, we addressed three questions: (1) does the morphology of basal dendrites in layers II/III reveal more branching in PFC than in MSV; (2) are there differences in the morphology of basal dendrites in layers II/III within PFC, between BA 10 and BA 11; and (3) are the basal dendrites of supragranular pyramidal neurons more complex than the basal dendrites in the infragranular pyramidal neurons? Although the present sample is small, due to a combination of the scarcity of postmortem tissue and the capriciousness of the Golgi technique, insights into the neural phenotype of this rare disorder can assist with the exploration of possible mechanisms leading to neurodevelopmental disorders (Chailangkarn et al., 2016) and can provide a platform for questions pertaining to the neural underpinning of human sociality and the evolution of the brain, specifically the PFC.

## Materials and methods

### Subjects

The morphology of pyramidal neurons was examined in the postmortem brain tissue of two adults with Williams Syndrome (WS). WS1 was a 31-year-old male, and WS 6 was a 47 year-old-male. Both died of cardiorespiratory arrest. WS diagnosis was established based on the Diagnostic Score Sheet (DSS) for WS subjects and, for WS 6, the diagnosis was further confirmed with fluorescent *in situ* hybridization (FISH) probes for elastin (ELN), which revealed hemizygous deletion of the elastin gene. The subjects were not diagnosed with any other conditions besides WS, although WS 6 was reported to have had an ischemic stroke, and to have suffered from aphasia and some agraphia. Both brain specimens were harvested within a postmortem interval of 18–30 h and had been kept in 10% formalin for up to 20 years. Given the long fixation time of our specimens, we have utilized the Golgi-Kopsch method (see below) suitable for specimens kept in formalin for 15 months or longer (Rosoklija et al., 2003). This method has been previously successfully used for analysis of dendritic morphology in human samples (Jacobs et al., 2003). Special attention was paid to factors influencing the outcome of methods involving silver crystals, such as temperature, exposure to light, and agitation (Rosoklija et al., 2003), which were kept constant in all of our specimens.

### Anatomic delineations of regions of interest

Tissue was sampled from the following cortical areas in the left hemisphere in a manner consistent to previous studies on TDs: frontal pole (BA 10), orbitofronal cortex (BA 11), primary somatosensory cortex (BA 3-1-2), primary motor cortex (BA 4), and secondary visual area (BA 18; both hemispheres to allow for adequate number of Golgi-impregnated neurons). Blocks from PFC were removed from the rostral part of the frontopolar gyrus in case of BA 10, and from the most rostral portion of the lateral orbital gyrus for BA 11. BA 3-1-2 and BA 4 were sampled from adjacent regions of the post- and pre-central gyri, representing the arm/hand region. For BA 18, the sample was located ~1.4 cm superior to the inferior surface of the occipital lobe and 2 cm from the midline. From each region of interest (ROI), a cortical block of 5 mm^3^ was used for Golgi processing.

### Tissue processing and morphological analysis of neurons

Cortical samples were processed using a modified Golgi-Kopsch technique (Jacobs et al., 2003), which appears effective for tissue stored in formalin for a prolonged period (Riley, 1979). The blocks sampled from each ROI were immersed in a 3% potassium-dichromate, 0.5% formalin solution and kept at 28°C for 8 days. The blocks were then transferred into 0.75% silver nitrate for 2 days and sectioned on a vibratome at a thickness of 120 μm. The sections were cut in 100% ethyl alcohol and transferred briefly into methyl salicylate, followed by toluene, mounted onto glass slides and cover-slipped.

In order to enable identification of the position within cortical layers for each traced neuron, adjacent blocks from each ROI were sectioned at 60 μm and stained for Nissl. These sections were used for a cytoarchitectonic analysis of the cortex in WS (Lew et al., 2017), and allowed for measurements of cortical laminar boundaries relative to the pial surface. In the Golgi stained sections, we measured the depth of the cell body relative to the pial surface and thus were able to distinguish neurons from supra- (layers II/III) and infra-granular (layers V/VI) layers.

**Figure 1:**
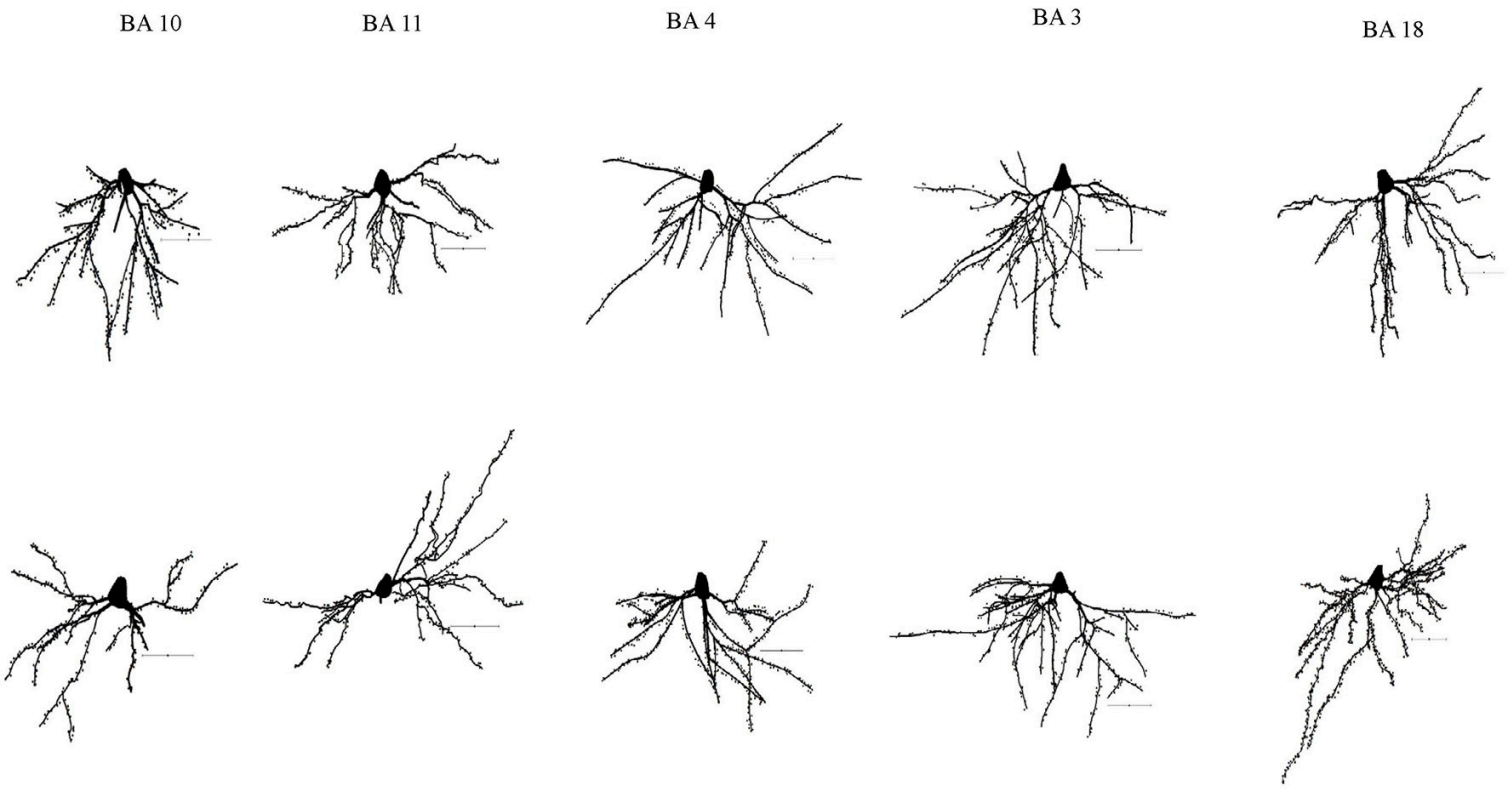
Representative tracings of cortical neurons from one subject (WS1) included in the present study. Scale bar: 50 μm.

Analysis of dendritic morphology was conducted only on neurons that displayed fully impregnated somata and three or more basal dendrites with at least third order dendritic branching (cf. Jacobs and Scheibel, 1993; Jacobs et al., 1997; Figure 1). The Golgi Kopsch method gave adequate staining mostly on basal dendrites; for this reason, and following Jacobs et al. (2001) in their analysis of basal dendritic morphology in typical subjects, we have limited our analysis to basal dendrites. Some of the analyzed dendrites displayed incomplete endings. They were included if they otherwise displayed the criteria for inclusion as outlined above (as recommended by Uylings et al., 1986), since including only neurons with dendritic arbors entirely contained within 120 μm thick sections biases the sample toward smaller neurons. All neurons included were oriented with the apical dendrite perpendicular to the pial surface; inverted and horizontal pyramidal cells were not analyzed. The analysis was conducted on basal dendrites in a total of 61 neurons from supragranular layers (between 1 and 10 neurons/ROI; Table 1). An additional 28 neurons from infragranular layers were successfully Golgi-impregnated and analyzed for the comparison of basal dendritic morphology between layers. Based on the success of the Golgi-Kopsch method, we were able to obtain sufficient number of neurons in the supragranular layers to statistically analyze basal dendritic morphology across cortical areas, and to compare infragranular layer neurons in all MSV areas combined to both PFC areas combined.

**Table 1:** Number of neurons included in this study (^*^includes both hemispheres).

**Subject**	**BA 10**	**BA 11**	**BA 4**	**BA 3**	**BA 18**	**Total**
WS 1 (layersII/III)	9	3	6	7	10^*^	35
WS 1 (layers V/VI)	1	2	5	1	3^*^	12
WS 6 (layers II/III)	2	7	8	8	1	26
WS 6 (layers V/VI)	0	3	3	8	2	16

Neuronal morphology was quantified along x-, y-, and z-coordinates using “Live Image” option on Neurolucida v.10 software (MBF Bioscience, Williston, VT) connected to Nikon Eclipse 80i microscope, with 40x(0.75) Plan Fluor dry objective. As the application of Sholl's concentric spheres or Eayrs' concentric circles for the analysis of neuronal morphology is not recommended when analyzing in three-dimensions (Uylings et al., 1986), we conducted dendritic tree analysis and included to following variables (cf. Jacobs and Scheibel, 1993; Jacobs et al., 2001): (1) total dendritic length (TDL)—summed length of all basal dendrites/neuron; (2) dendritic segment count (DSC)—total number of basal dendritic segments/neuron; (3) dendritic spine number (DSN)—total number of dendritic spines/neuron; (4) mean segment length (MSL)—mean length of basal dendrite/neuron (calculated as TDL/DSC); (5) mean segment count (MSC)—mean number of segments/100 μm of basal dendritic length.

All tracings were conducted by the same investigator (Branka Hrvoj-Mihic), blind to the diagnosis and ROI. Intrarater reliability was assessed by having the tracer trace the same neuron after a period of time. The average coefficient of variation between the results of retraced neurons was 2% for TDL and DSC, and 3% for DSN. The accuracy of the tracings was further checked by having three raters (Branka Hrvoj-Mihic, Bob Jacobs, Lisa Stefanacci) trace the same neuron.

### Statistical analyses

Statistical analyses were conducted to address three lines of investigation: (1) variation in the morphology of basal dendrites across cortical areas in supragranular layers in each subject (WS 1, WS 6); (2) differences in the morphology of basal dendrites between PFC (BA 10 and BA 11) and MSV (BA 4, BA 3-1-2, BA 18) in each subject; and (3) comparison of dendritic morphology between supra- and infra-granular layers in high-integration (PFC; BA 10 and BA 11) versus MSV (BA 4, BA 3-1-2, BA 18) cortical areas in each subject.

Variation in basal dendritic morphology across cortical areas was analyzed using single-factor ANOVA. For the analysis of dendritic morphology between supra- and infragranular layers, as well as between combined PFC and MSV areas, the data were analyzed using unpaired Student's *t*-test with Welch correction or, in cases where the data did not display a normal distribution, using Mann-Whitney test. All analyses were performed on Prism v.7 (GraphPad Software, Inc.). Since the Golgi modification used in this study differed from the modifications used in studies examining morphology of dendrites in TDs (Golgi-Kopsch versus rapid/Golgi Cox; Jacobs et al., 2001; Petanjek et al., 2008), we were not able to directly compare absolute values across studies. Instead, our analysis focused on relative differences in basal dendritic length and branching within different cortical areas and layers within each WS subject.

## Results

### Morphology of basal dendrites in supragranular cortical layers in WS

**Figure 2:**
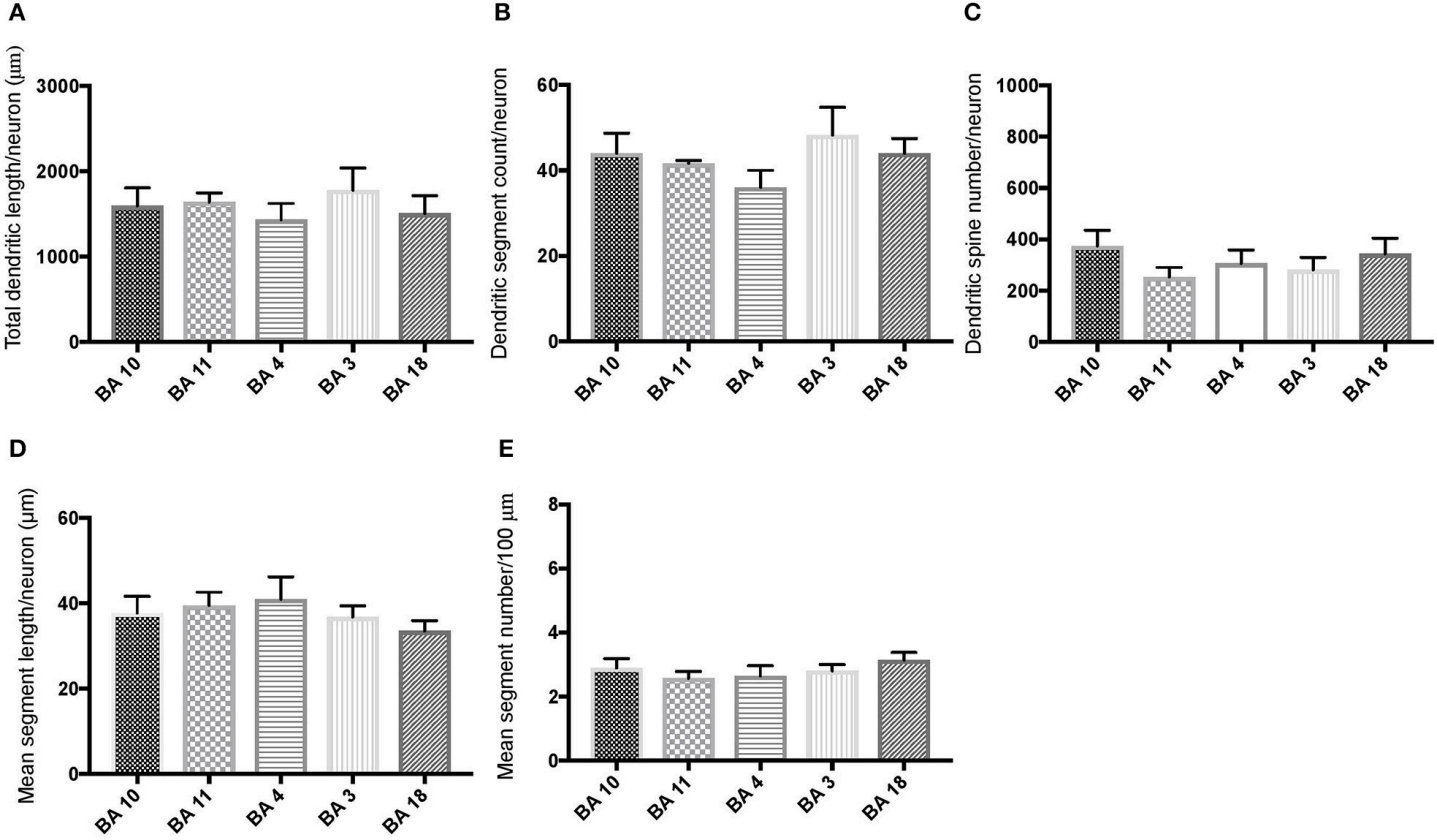
Bar graphs representing basal dendritic variables in supragranular layers (layers II/III) across cortical areas in subject WS 1 (31 year-old, male): **(A)** total dendritic length/neuron (TDL, μm); **(B)** dendritic segment count (DSC); **(C)** number of dendritic spines (DSN); **(D)** mean segment length (MSL, μm), and **(E)** mean number of dendritic segments (MSC). Data are presented as mean ± SEM.

**Figure 3:**
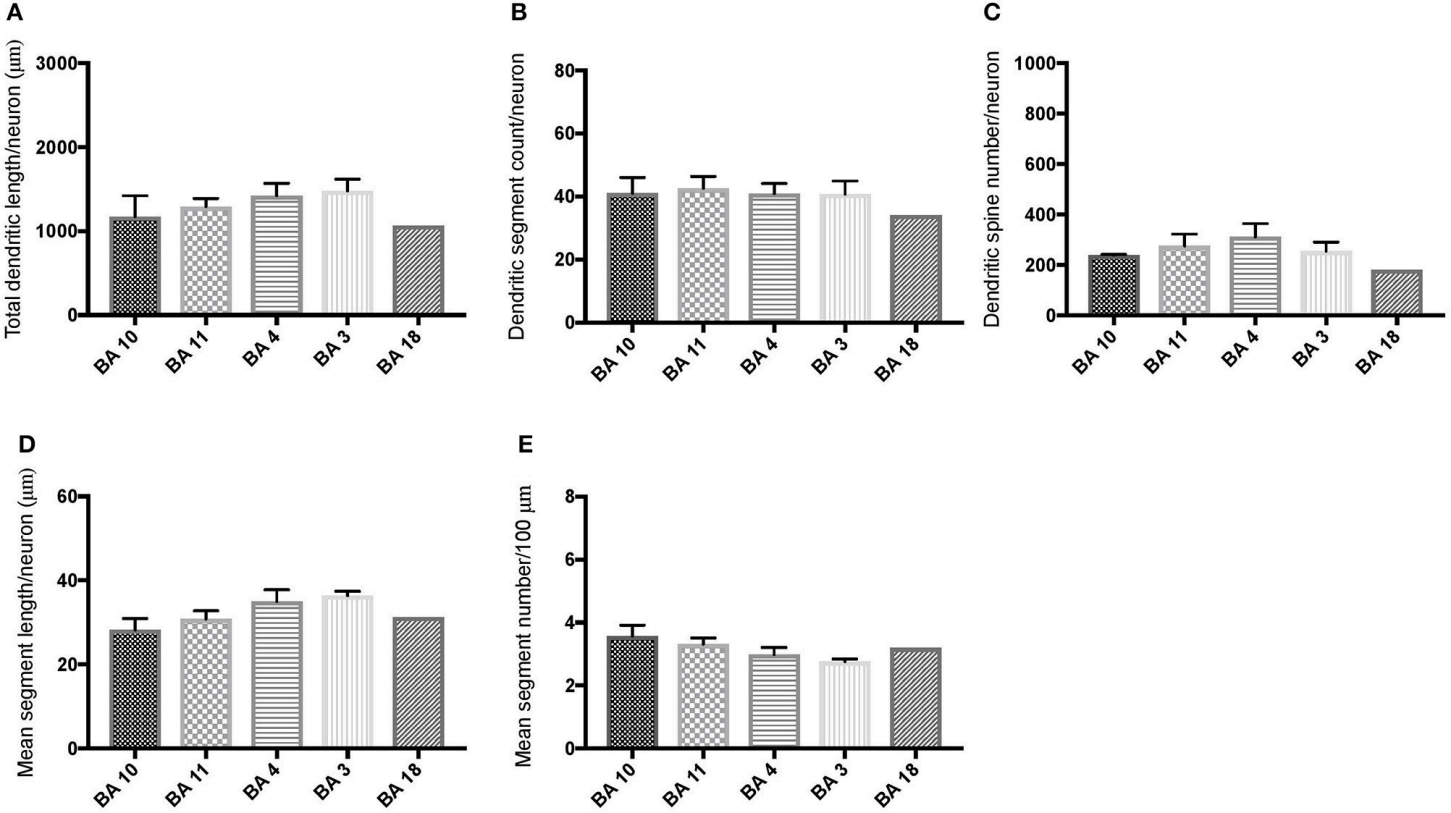
Bar graphs representing basal dendritic variables in supragranular layers (layers II/III) across cortical areas in subject WS 6 (47 year-old, male): **(A)** total dendritic length (TDL, μm); **(B)** dendritic segment count (DSC); **(C)** number of dendritic spines (DSN); **(D)** mean segment length (MSL, μm), and **(E)** mean number of dendritic segments (MSC). Data are presented as mean ± SEM.

**Figure 4:**
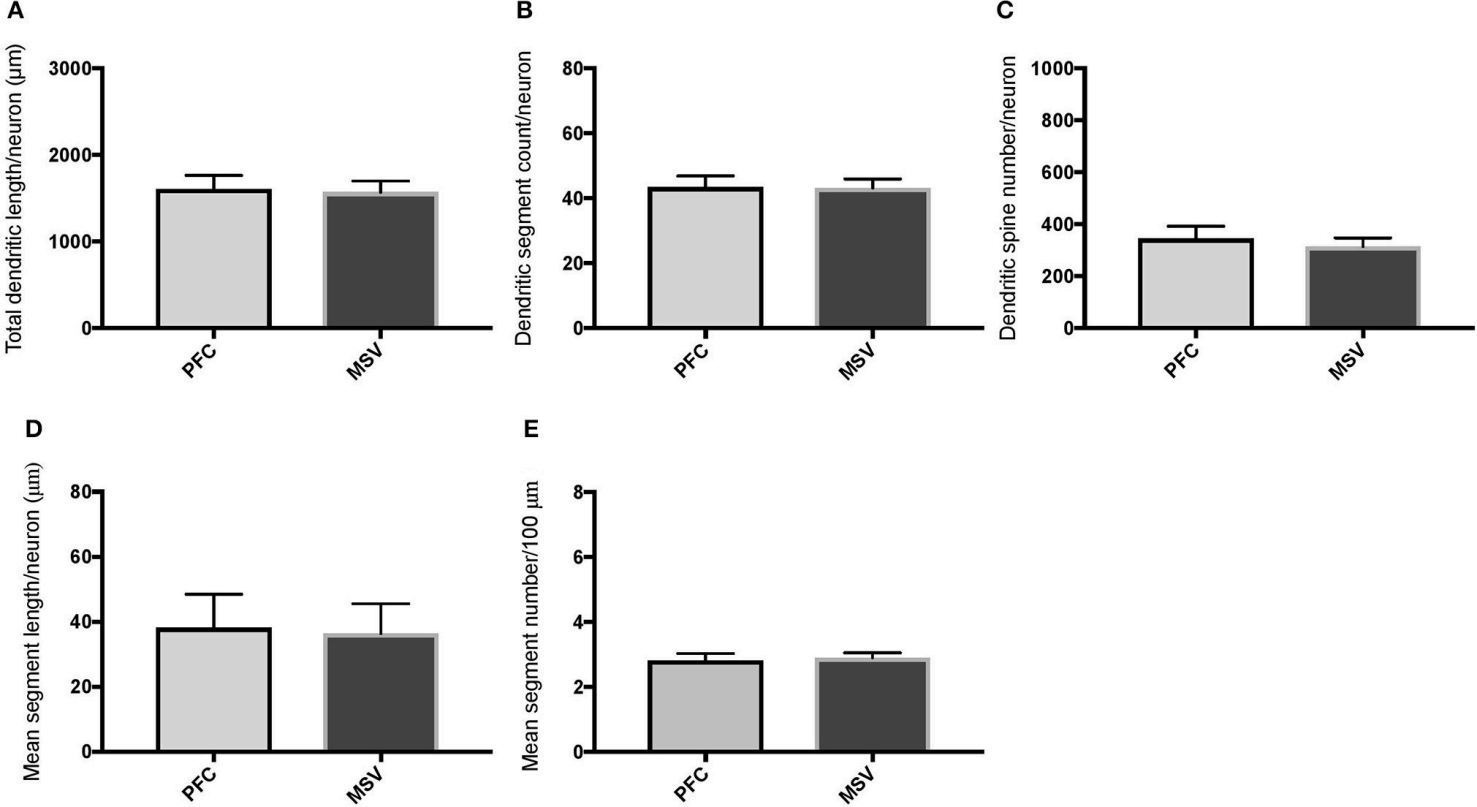
Bar graphs comparing basal dendritic variables in supragranular layers (layers II/III) of the prefrontal cortex (BA 10, BA 11) and motor-sensory-visual areas examined (MSV; BA 4, BA 3, BA 18) in subject WS 1 (31 year-old, male): **(A)** total dendritic length (TDL, μm); **(B)** dendritic segment count (DSC); **(C)** number of dendritic spines (DSN); **(D)** mean segment length (MSL, μm), and **(E)** mean number of dendritic segments (MSC). Data are presented as mean ± SEM.

**Figure 5:**
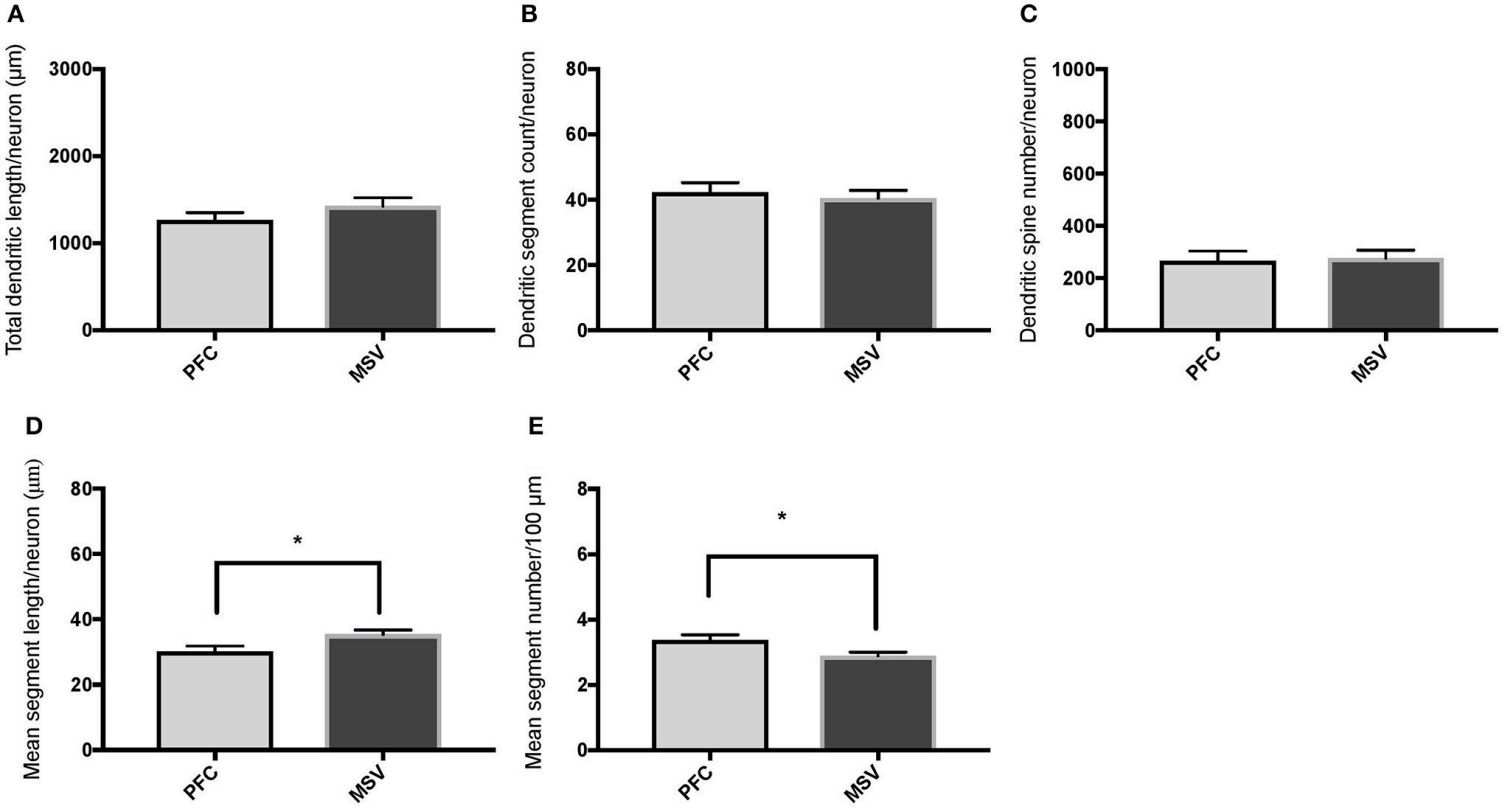
Bar graphs comparing basal dendritic variables in supragranular layers (layers II/III) of the prefrontal cortex (BA 10, BA 11) and the motor-sensory-visual areas examined (MSV; BA 4, BA 3, BA 18) in subject WS 6 (47 year-old, male): **(A)** total dendritic length (TDL, μm); **(B)** dendritic segment count (DSC); **(C)** number of dendritic spines (DSN); **(D)** mean segment length (MSL, μm), and **(E)** mean number of dendritic segments (MSC). Data are presented as mean ± SEM. ^*^P ≤ 0.05.

Comparison of basal dendritic morphology in supragranular pyramidal neurons revealed no statistically significant difference in dendritic/spine measures for any of the individual cortical areas, either in WS 1 or WS 6 (Figures 2, 3). When the values for the two PFC areas—BA 10 and BA 11—were grouped together and analyzed against MSV areas (BA 4, BA 3-1-2, BA 18) combined, the difference between PFC and MSV reached statistically significant difference in WS 6. Specifically in WS 6, MSL values were significantly higher for MSV areas compared to the two PFC areas combined (*P* = 0.02) and the reverse was the case for MSC, with values higher in PFC (*P* = 0.03; **Figures 5D,E**). The other measures did not reveal any statistically significant differences (Figures 4, 5A–C).

### Comparison of basal dendrites between supra- and infragranular layers

We also examined pyramidal neurons in infragranular cortical layers in order to compare the basal dendritic morphology in supra- versus infragranular layers for PFC (BA 10 and BA 11) versus the three MSV areas (BA 4, BA 3-1-2, BA 18) combined. In PFC of WS 6, TDL and DSC values were higher in supragranular than in infragranular layers, reaching statistical significance (TDL *P* = 0.02 and DSC *P* = 0.007; **Figures 7A,B**). No statistically significant differences were observed in WS 1 PFC, but all variables analyzed (except for DSN) pointed in both subjects either in the same direction with higher values in supra- relative to infragranular layers, or showed little difference between the layers.

**Figure 6:**
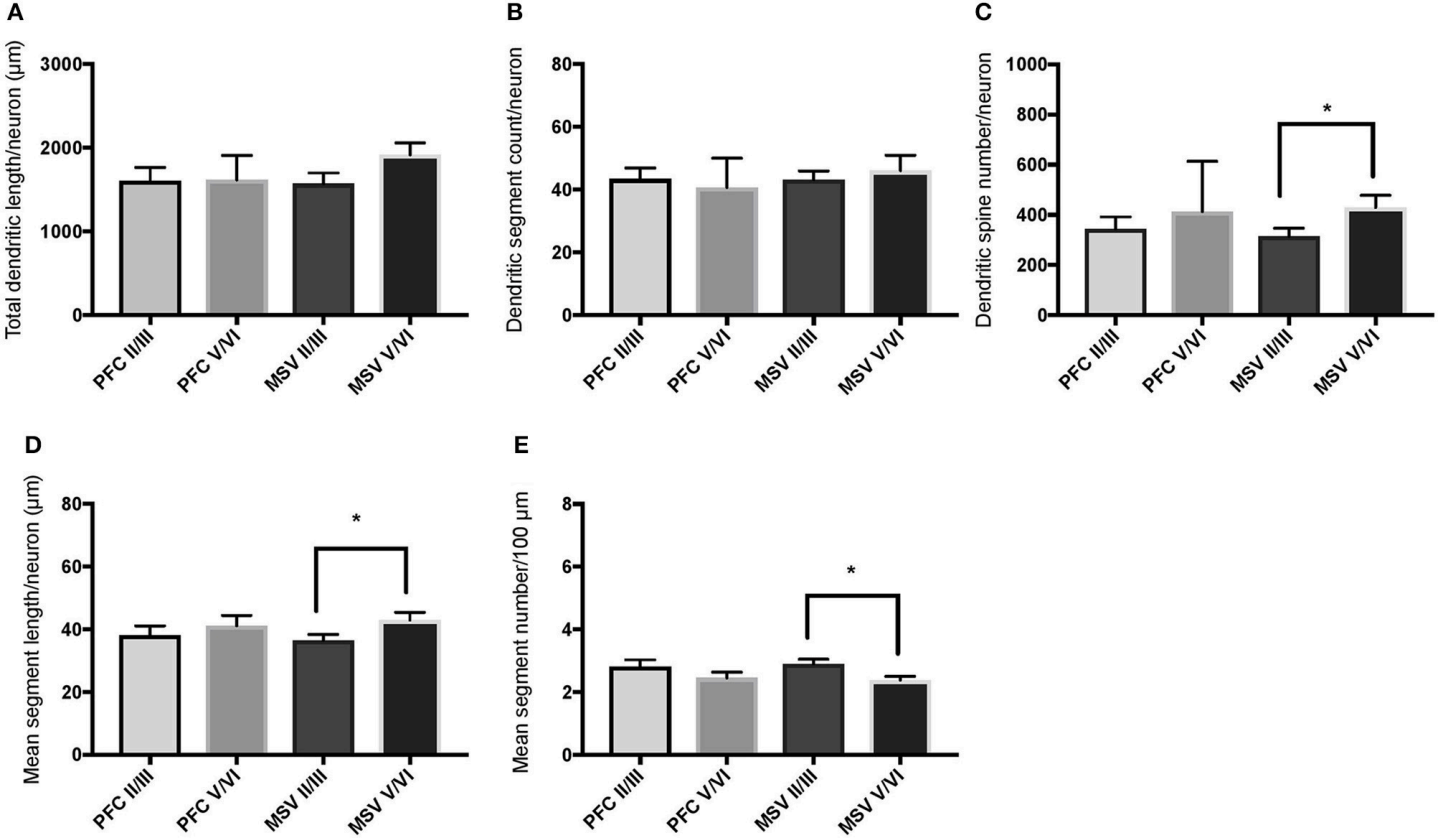
Bar graphs illustrating the relationship in basal dendritic morphology between supra- (II/III) and infra-granular layers (V/VI) in PFC and motor-sensory-visual areas (MSV; BA 4, BA 3, BA 18) in the subject WS 1 (31 year-old, male): **(A)** total dendritic length (TDL, μm); **(B)** dendritic segment count (DSC); **(C)** number of dendritic spines (DSN); **(D)** mean segment length (MSL, μm), and **(E)** mean number of dendritic segments (MSC). Data are presented as mean ± SEM; ^*^P ≤ 0.05.

**Figure 7:**
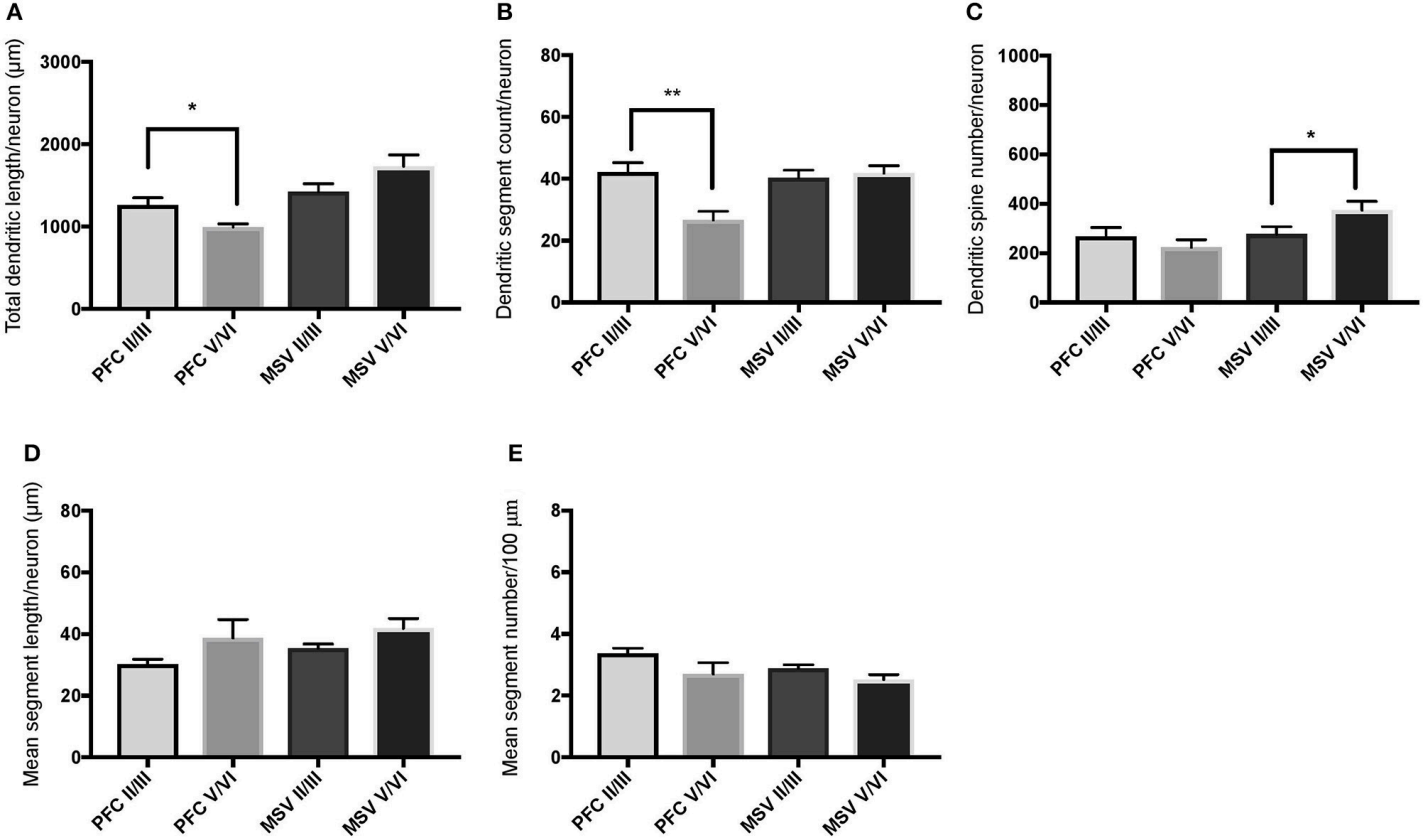
Bar graphs illustrating the relationship in basal dendritic morphology between supra- (II/III) and infra-granular layers (V/VI) in PFC and motor-sensory-visual areas (MSV; BA 4, BA 3, BA 18) in the subject WS 6 (47 year-old, male): **(A)** total dendritic length (TDL, μm); **(B)** dendritic segment count (DSC); **(C)** number of dendritic spines (DSN); **(D)** mean segment length (MSL, μm), and **(E)** mean number of dendritic segments (MSC). Data are presented as mean ± SEM; ^*^P ≤ 0.05, ^**^P ≤ 0.01.

In the MSV areas of both subjects, TDL was higher in infragranular than in supragranular layers, with a similar trend toward significance (*P* = 0.08 in both WS 1 and WS 6; Figures 6A, 7A), and DSN was significantly higher in infragranular layers (WS 1 *P* = 0.05 Figure 6C; WS 6 *P* = 0.02 Figure 7C). Also in the MSV areas, values of all variables, even if not reaching statistical significance, were higher in infragranular versus supragranular layers (Figures 6, 7). The only exception was MSC with supragranular layers higher than infragranular layers in both subjects, reaching statistical significance in WS1 (*P* = 0.01; Figure 6E). It is important to note that the absolute values for MSC differed little between unimodal areas of WS 1 and WS 6; however, only in WS 1 the difference reached statistical significance.

## Discussion

Analysis of WS pyramidal neurons revealed differences in the organization of basal dendrites in WS when compared to TDs (Jacobs et al., 1997, 2001) or to other pathologies with compromised social functioning, including ASD and RTT (Belichenko et al., 1994; Hutsler and Zhang, 2010; see below). The differences were layer-specific, more prominent in PFC areas, and displayed an overall pattern of dendritic organization that sets WS apart from other disorders.

### Organization of basal dendrites in supragranular layers across the cortex and within PFC

In analyzing supragranular cortical layers, we focused specifically on the difference in dendritic length (TDL, MSL) and branching (DSC, MSC) in PFC areas compared to MSV (BA 4, BA 3-1-2, BA 18). Our question was whether the morphology of basal dendrites in supragranular layers would reveal more branching in PFC than in MSV areas in WS.

Based on prior reports of TD data (Jacobs et al., 2001), we expected that basal dendrites in supragranular layers would be longer, exhibit more dendritic spines, and increase in complexity from cortical areas BA 3-1-2, through BA 4 and BA 18, and be the most complex in the two PFC areas (BA 11 and BA 10). Nevertheless, our results revealed no statistically significant difference in dendritic length (TDL, MSL) or dendritic branching (DSC, MSC) of basal dendrites between each of the cortical areas examined, or even a general pattern pointing in such a direction, in either WS 1 or WS 6 (Figures 2, 3). For most variables in one or both subjects, PFC values were actually either below or about the same as MSV values, for each of BA 3-1-2, BA 4, and BA 18. This lack of dendritic variation between PFC and rest of cortical areas in supragranular layers represents another finding that has not been reported for most other disorders, with the exception of a similar pattern in RTT (Belichenko et al., 1994), another disorder with compromised social functioning.

In each WS subject, basal dendrites in one or more of the MSV areas emerged as having the longest and most branched basal dendrites relative to the rest of the cortical areas examined. In WS 1 for example, this was seen in BA 3-1-2 (Figures 2A,B), whereas in WS 6, it was seen in both BA 3-1-2 and BA 4 (Figure 3A). However, this pattern of longest and most branched dendrites in one MSV area disappeared when the mean values of segment length (MSL) and segment count (MSC) were taken into account (Figures 2D,E, 3D,E). Despite the lack of statistical significance, these findings are of interest given that they were found in both subjects in our sample, while a similar pattern has not been reported in TD or in other disorders.

The functional implications of our observations are difficult to interpret, but it can be suggested that processing of one modality may be emphasized in WS, at the expense of stimulus evaluation and response choice in high-integration areas of the PFC. Partial support for the view that multi-modal integration is compromised in WS comes from fMRI research, which suggests that WS subjects display less activation in high-integration areas in the temporal lobe (superior and middle temporal gyri, and superior temporal sulcus) and increased activation in subcortical structures (Levitin et al., 2003) during music processing. Thus, it is possible that compromised integration and evaluation of different stimuli may be a general feature of cortex in WS.

With respect to whether there is variation within PFC, we expected based on TD findings (Jacobs et al., 1997, 2001) that BA 10 would display longer, more branched, and more spinous basal dendrites compared to BA 11. Nevertheless, we did not observe this relationship for TDL, DSN, MSL, and MSC. Instead, in each of our WS subjects, BA 10 and BA 11 differed little from one another (Figures 2A,B,D,E, 3A–E). Most studies examining neurodevelopmental disorders in PFC target a single cortical area (e.g., Vukšić et al., 2002; Table 2) and the ways in which different areas within the PFC may be affected in various disorders remain not well researched. It is intriguing that we did not find differences in basal dendritic complexity between BA 10 and BA 11 and that both areas displayed values equal or lesser than in MSV areas. Thus, compromised dendritic length and branching may not be limited to a single PFC area, but could instead represent a shared feature of neural systems involving the PFC as a whole. It is important to note that the frontal pole (BA 10) and the orbitofrontal cortex (OFC; for example BA 11) are part of distinct neural systems (Barbas, 2007), with BA 11 being part of socioemotional circuitry, while BA 10 is implicated in higher-order cognitive tasks (Stuss and Benson, 1984). Existing MRI studies report the presence of structural differences in frontal lobes between WS and TD (Reiss et al., 2000). More specifically, OFC has been previously reported as structurally different in WS (Meyer-Lindenberg et al., 2004) and, functionally, not activated in the same social situations (e.g., threatening faces) compared to TD, unlike the dorsolateral and mesial PFC (Meyer-Lindenberg et al., 2005). Given that the present study focused on two distinct areas of PFC at a resolution not possible for macroscopic studies and found no differences in the organization of basal dendrites between BA 10 and BA 11, it is possible that compromised social functioning specific to WS—characterized by lack of social inhibition, heightened desire to interact with strangers, and failure to follow the complex norms guiding social behavior (Karmiloff-Smith et al., 1995)—is not limited to OFC but, instead, is underlined by compromised morphology in a number of areas within PFC.

**Table 2:** List of selected postmortem human studies examining morphological changes on dendrites of cortical pyramidal neuron including PFC.

**Areas examined**	**Pathology**	**Age**	**Subject numbers, diagnosis**	**References**
Frontal cortex Parietal cortex Temporal cortex	ASD	12, 27 years	4 ASD	Williams et al., 1980
Prefrontal cortex (BA 9) Temporal cortex (BA 21) Parietal cortex (BA 7)	ASD	10–45 years	10 ASD 15 controls	Hutsler and Zhang, 2010
Prefrontal (BA 10) Frontal (BA 4) Temporal (BA 21)	RTT	16–24 years	2 RTT 11 TRPE 6 controls	Belichenko et al., 1994
Frontal cortex Temporal cortex Occipital cortex	RTT/DS	6–35 years	16 RTT 6 DS 9 controls	Armstrong et al., 1998
Prefrontal (BA 9)	DS	Newborn, 2.5 month	2 DS 2 controls	Vukšić et al., 2002
Temporal pole (BA 38) Temporal cortex (BA 22/21)	Schizophrenia	Adult	13 schizophrenia 1 schizophrenia-like psychosis 11 controls	Garey et al., 1998
Frontal cortex (BA 46) Occipital cortex (BA 17)	Schizophrenia	Adult	15 schizophrenia 15 non-schizophrenia with psychiatric illness 15 controls	Glantz and Lewis, 2000
Mesial frontal lobe Temporal lobe	Epilepsy	Not reported	14	Vaquero et al., 1982
Motor cortex (BA 4) PFC (BA 10) Broca's area (BA 44)	Callosotomy	Adult	2 callosotomy	Jacobs et al., 2003

*ASD, autism spectrum disorder; RTT, Rett syndrome; DS, Down syndrome; TRPE, therapy resistant partial epilepsy*.

### Comparison of basal dendritic branching between supra- and infragranular layers in PFC and MSV in WS cortex

With respect to our last question, the aim was to determine whether WS neurons display differences in branching and length of basal dendrites between supra- and infragranular layers as reported for the PFC in TDs (Petanjek et al., 2008), with dendrites of the neurons in supragranular layers being longer than those on the neurons in infragranular layers. The results indicated that dendritic length and branching across layers in PFC of WS were similar to TDs (Petanjek et al., 2008). As with TDs, there is individual variation, with differences being significant for some of the parameters in one subject (WS 6; Figures 7A,B), while a trend toward longer and more branched basal dendrites was present in the other subject (WS 1; Figures 6A,B). Although the TD study sampled exclusively magnopyramidal neurons from lower layer III (Petanjek et al., 2008), and in contrast, the present study was based on neurons throughout the depth of layer III, a similar pattern was observed here for WS. These findings suggested that the relative degree of branching as defined by the length (TDL) and segment number (DSC) of basal dendrites in supra- and infragranular layers of PFC may have been preserved in WS.

It is of interest that neuronal body density in layer V/VI of PFC (specifically BA 10) is decreased in WS compared to TDs, whereas no significant differences in density were observed in layer II/III (Lew et al., 2017). Based on this finding, there may be an increase in soma size or glia numbers and/or increased length and branching of pyramidal neurons in infragranular layers of BA 10 in WS. The present findings on PFC provided partial argument against the latter possibility, namely that a decrease in neuronal density is accompanied by an increase in dendritic length and branching in layers V/VI of BA 10. It is also of interest that, when the morphology of basal and apical dendrites of layers V/VI WS neurons in MSV areas was directly compared to TDs (Chailangkarn et al., 2016), dendrites in WS emerged as longer and more branched. Given that neuronal soma density is also increased—although not statistically significant—in BA 4, BA 3-1-2, and BA 18 in WS (Lew et al., 2017) it can be preliminary suggested that the networks subserving processing of motor, sensory, and visual stimuli through the infragranular layers are emphasized in WS. The conclusion is further supported by the findings from the analysis of dendritic branching, especially with the results related to the increased length of basal dendrites compared to supra-granular layers (Figures 6, 7), more complex morphology of apical and basal dendrites compared to TD in layer V/VI (Chailangkarn et al., 2016), and longer and more branched dendrites in layer V/VI of MSV compared to layer V/VI in PFC (Figures 7A,B).

The current findings suggest that the pattern of basal dendritic complexity in WS differs from that described in TDs. It has been suggested that in TD, an increase in dendritic length tends to correspond with higher values for dendritic segments (i.e., branching Jacobs et al., 2001; Petanjek et al., 2008). In WS, this pattern seems to be reversed, and to differ across cortical areas, with PFC neurons being on average shorter and more branched, and the basal dendrites in MSV areas longer, but less branched. Although this finding is difficult to interpret, it may reflect regional developmental differences between PFC and MSV areas. Namely, pyramidal cell dendritic systems in primary processing areas appear to mature earlier than in the PFC (Marin-Padilla, 1970; Mrzljak et al., 1990; Koenderink et al., 1994; Travis et al., 2005; Petanjek et al., 2008). During postnatal maturation, basal dendrites undergo an extensive period of growth, branching, and spinogenesis. Analyses of maturation of the layer III magnopyramidal neurons in PFC suggest that the majority of dendritic maturation occurs during the first two and a half years, characterized by two growth spurts: from birth to 2.5 months postnatal, and from 16 months until 2.5 years (Petanjek et al., 2008). Whereas dendritic length increases in both of these periods, the first maturation period is characterized by an increase in dendritic segments, whereas the increase in length observed during the second period results mostly from increase in length of dendritic segments, not by an increase in the number of segments (Petanjek et al., 2008). If the factors underlying the pathologies on basal dendrites in WS do not occur immediately after birth—the period when dendrites in primary processing areas are undergoing increases in length and PFC dendrites mostly in branching—the aspects of the morphology developing perinatally would be spared. This would result in longer dendrites in primary processing (MSV) areas and more branched basal dendrites in the PFC. This is the pattern observed here in the cortex of WS subjects. Additional studies analyzing the timing of the activity of genes deleted in WS could help evaluate this hypothesis, and the role of specific developmental time-periods, which may be crucial for the appearance of dendritic morphology in WS.

Alternatively, since the morphology of dendrites is influenced by synaptic activity (Rajan and Cline, 1998), it has been suggested that dendritic pathologies associated with various disorders may represent a neuron's attempt to supplement a lack of inputs by increasing the number of connections, inferred based on an increase in dendritic length, dendritic branching, or in the number of dendritic spines (reviewed by Fiala et al., 2002; Srinivasan et al., 2014). In WS, areas with varying integrative abilities may have solved this problem differently, by increasing dendritic length in the case of MSV areas, or increasing the number of branches in PFC neurons. In the early-maturing MSV regions, the increase in connectivity may have been achieved by increasing the basal dendritic length, whereas the same task may be achieved in later-maturing PFC by increasing the branching capacity.

It has been proposed that specific developmental features of the PFC may make the dendrites on the pyramidal neurons in PFC more prone to modifications in neurodevelopmental disorders (Penzes et al., 2011). As previously mentioned, in the disorders studied pre- and post-natally, differences in the morphology of basal dendrites were found during the perinatal period (Marin-Padilla, 1972, 1976; Jay et al., 1990), suggesting that they could result from neurons' inadequate response to received inputs. A similar hypothesis was previously proposed for abnormalities in dendritic spines in neurological disorders (Fiala et al., 2002), which interpreted findings on the spine number and morphology in disorders as the improper establishment of synaptic inputs. However, since inputs into a neuron are determined by the length of dendrites, the extent of dendritic branching, and the number and density of dendritic spines—all of which influence integrative properties of dendrites and response to the inputs (Poirazi and Mel, 2001; Srinivasan and Stevens, 2011)—it is important to take all these aspect of dendritic morphology into account when drawing conclusions about functional implications or potential causes behind the morphology of dendrites in developmental disorders.

### Findings from WS and implications for understanding unique features of the human brain

Among the areas we examined in WS, PFC has received special interest from an evolutionary perspective. In human evolution, the PFC has been reorganized with some areas having become enlarged (BA 10) and others having decreased in size disproportionately (BA 13; Semendeferi et al., 1998, 2001). Specific PFC areas, such as BA 10, have also undergone a microstructural reorganization, including changes in the number and spatial distribution of neurons (Semendeferi et al., 2001, 2011), organization of basal dendrites (Bianchi et al., 2012), and maturation (Sakai et al., 2011). These differences in neuronal organization between humans and great apes seem to be especially prominent in cortical layer III (Semendeferi et al., 2011), suggesting an emphasis on information processing in BA 10 and the functional demands placed specifically on layer III.

In addition to humans (Jacobs et al., 2001), dendritic branching has been examined in the cortex of other primate species: the galago *(Otolemur garnetti)*, owl monkey *(Aotus trivirgatus)*, vervet monkey *(Cercopithecus pygerythrus)*, marmoset *(Callithrix jacchus)*, and baboon (*Papio ursinus*; Elston et al., 2006 and the references therein) and the common chimpanzee (*Pan troglodytes*; Bianchi et al., 2012). In all of these species, pyramidal neuron basal dendrites in PFC displayed more complex morphologies—defined as either higher values for basal dendritic field area (Elston et al., 2006) or higher TDL and DSC (Jacobs et al., 2001; Bianchi et al., 2012)—compared to primary processing and unimodal areas. The number of dendritic spines is also higher in the PFC (Jacobs et al., 2001; Elston et al., 2006; Bianchi et al., 2012) and, at least in humans and monkey species, the increase in DSN tended to be associated with the absolute size of the cortical regions, and not the size of a basal dendritic area (Elston et al., 2006). These comparative cross species studies suggest that an increase in complexity of basal dendrites in PFC may represent a feature shared by all primates (but see also data for the African elephant; Jacobs et al., 2011) and that organization of basal dendrites (basal dendritic field area or TDL and DSC) and the number of dendritic spines represent two aspects of pyramidal cell anatomy that respond differently to the computational demands of the cortical circuitry they form. It has been suggested that the need to navigate complex and hierarchical social environments represented selective pressures influencing changes in the human brain during the evolution (Humphrey, 1976; Byrne and Whiten, 1989; Dunbar, 1998). Studies examining these areas in neurological disorders can further our understanding on the connection between anatomy, function, and specialization of cortical areas in humans.

## Conclusions

In the present study, we explored the morphology of basal dendrites in WS—a disorder with distinct genetic and behavioral manifestations—with attention to PFC and the variation across cortical layers and cortical areas. We found that pathologies in the organization of basal dendrites in WS depend on the cortical area and layers examined, with most of the differences with TD found in supragranular layers and in the PFC. Thus, it can be concluded that the behavioral phenotype seen in WS may, at least in part, be influenced by alterations in the morphology of basal dendrites. It would be important in the future to combine the present findings with additional analyses of neuron density in the cortex, as well as with analyses of dendritic branching in the subcortical areas projecting to different areas in PFC. Such studies will provide further insight into organization of dendrites across several cortical areas in WS, a unique disorder that can offer valuable insights into the interplay between genes, brain and behavior.

## Author contributions

KS and LS conceived and designed the experiments. BHM, KLH, and CHL processed the tissue. BHM collected the data, and analyzed the results and wrote the manuscript with KS and with the input from all authors. UB and BJ contributed to the overall conception and design of the research.

### Conflict of interest statement

The authors declare that the research was conducted in the absence of any commercial or financial relationships that could be construed as a potential conflict of interest.
